# Prefabrication of a functional bone graft with a pedicled periosteal flap as an *in vivo* bioreactor

**DOI:** 10.1038/s41598-017-17452-5

**Published:** 2017-12-21

**Authors:** Ru-Lin Huang, Mathias Tremp, Chia-Kang Ho, Yangbai Sun, Kai Liu, Qingfeng Li

**Affiliations:** 1 Department of Plastic and Reconstructive Surgery, Shanghai Ninth People’s Hospital, Shanghai Jiao Tong University School of Medicine, 639 Zhizaoju Road, 200011 Shanghai, China; 2 Department of Plastic, Reconstructive, Aesthetic, and Hand Surgery, University Hospital Basel, Spitalstrasse 21, 4031 Basel, Switzerland

## Abstract

The *in vivo* bioreactor principle, which focuses on using the body as a living bioreactor to cultivate stem cells, bioscaffolds, and growth factors and leveraging the body’s self-regenerative capacity to regenerate new tissue, has been considered a potential approach for bone defect reconstruction. The histological characteristics of the periosteum allow it to possess a remarkable capacity to induce bone growth and remodeling, making it suitable as an *in vivo* bioreactor strategy for bone graft prefabrication. The present study was designed to prefabricate vascularized bone grafts using pedicled periosteal flaps and decellularized bone matrix (DBM) scaffolds in a rabbit model. The muscular pouches created in the femoral muscle were acted as a control. Our histological results revealed that both the periosteal flap group and muscular pouch group induced bone tissue formation on the DBM surface at both 8 and 16 weeks postoperatively. However, micro-computed tomography (microCT) scanning, biomechanical, and histomorphometric findings indicated that bone grafts from the periosteal flap group showed larger bone mass, faster bone formation rates, higher vascular density, and stronger biomechanical properties than in the muscular pouch group. We suggest that using the pedicled periosteal flap as an *in vivo* bioreactor is a promising approach for functional bone graft prefabrication.

## Introduction

Large volume bony defects resulting from traumatic incidents, congenital abnormalities, infection, or cancer resections represent a great challenge for orthopedic, craniomaxillofacial, and reconstructive surgeons. Historically, different strategies, including bone transport methods (distraction osteogenesis), implantation of biomaterials (natural and synthetic materials), or bone grafting (autografts and allografts), have been used for bone defect reconstruction, but none of the currently available bone substitutes has all the ideal characteristics^1^.

Bone tissue engineering (BTE), which aims to regenerate new biological bone tissue with similar properties and functions as natural occurring bone rather than building new spare parts^2,3^, has seen tremendous advances over the years and gives hope to solving this problem. Strategies of BTE have relied on one of two approaches: *in vitro* or *in vivo* tissue engineering. The *in vitro* BTE strategy attempts to create functional bone grafts by culturing osteogenic cells on bioactive scaffolds in an *in vitro* environment^3^. However, when *in vitro* engineered bone grafts are transferred *in vivo*, they lack the defined vascular and nerve networks to support cell survival and matrix synthesis and thus must rely on the ingrowth of neo-vascular structures from their surroundings, resulting in limited long-term outcomes in clinical therapeutic studies^4–6^. An emerging trend to circumvent these problems follows the *in vivo* bioreactor principle, which uses the body as a bioreactor to cultivate the traditional triad (scaffolds, cells, and growth factors) and to leverage the self-regenerative capacity of the body to regenerate new bone tissue. We previously used a vessel bundle in combination with the muscularis membrane to act as an *in vivo* bioreactor and wrap a BMSC-seeded beta tricalcium phosphate (β-TCP) construct in a rabbit model. The results, as demonstrated by new cartilage and bone tissue formation in the β-TCP construct, proved that it is possible to engineer a bone graft following the *in vivo* bioreactor principle^7^. To date, the *in vivo* bioreactor principle concept has been applied in clinical conditions for bone defect reconstruction^8–13^. Although limited clinical successes have been reported, this concept provides a promising and translatable approach for bone regeneration.

Different *in vivo* bioreactor strategies for regenerating bone tissue, such as the muscular pouch^14^, arteriovenous bundle (AVB)^15^, and arteriovenous loop (AVL)^16^, have been extensively investigated. Due to limited bone regeneration efficiency and clinical transition feasibility, research is ongoing to develop an ideal *in vivo* bioreactor strategy for clinical application. The periosteum is a thin but highly vascularized and innervated tissue with a bilayer structure, the outer fibrous layer and the inner cambium layer, which consists primarily of skeletal progenitor cells and osteoblasts that possess a remarkable capacity to allow appositional bone growth as well as cortical bone modeling and remodeling^17^. Considering these findings, surgeons have harnessed this osteogenic capacity to induce bone regeneration for more than two centuries. Tatara AM *et al*. generated large volumes of mineralized tissue for mandibular reconstruction using the rib periosteum in a sheep model^18^. However, current periosteum-based strategies are still limited by the lack of a large-sized periosteum donor site and a feasible surgical procedure for clinical translation.

The prefabrication technique is one of the most exciting areas in plastic surgery because of its bridging role between conventional reconstructive surgery and tissue engineering^19^. A typical prefabrication procedure begins with the implantation of a vascular pedicled flap to an avascular receipt territory/construct. After a period of *in vivo* cultivation, the vascular pedicled flap will revascularize and even regenerate new tissue in the receipt territory/construct, allowing the prefabricated new flap to be transferred to any specified recipient site, and greatly expanding the armamentarium of reconstructive options^20^. Therefore, we hypothesized that a functional bone graft could be regenerated by using a pedicled periosteal flap as an *in vivo* bioreactor and a DBM as a bioscaffold. Here, we here report our results in the form of histologic, microCT, and biomechanical review, and we discuss the feasibility of translating this promising bone graft prefabricating strategy into clinical conditions.

## Results

### Properties of the DBM scaffolds

**Figure 1:**
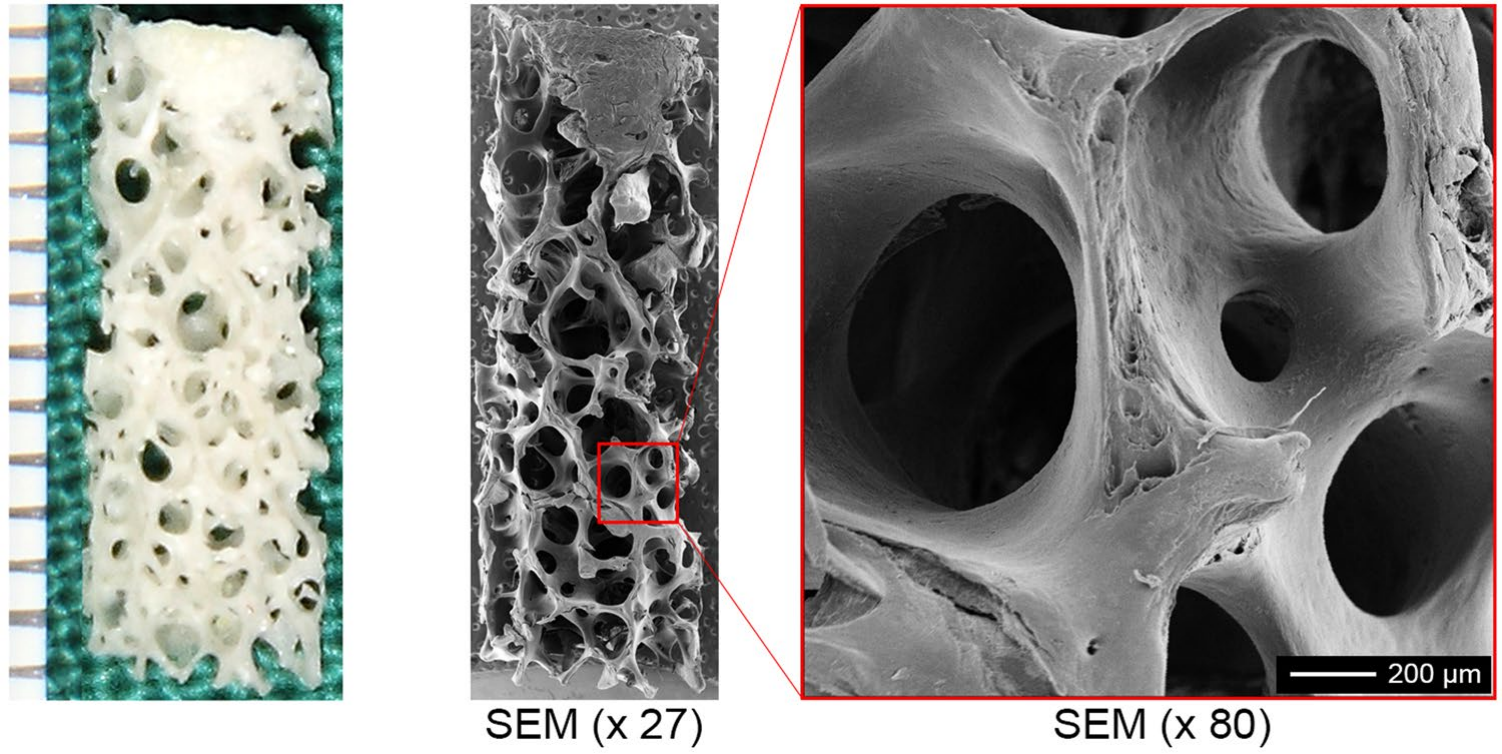
Gross view and SEM examination of the DBM scaffold. (**A**) Gross view of the DBM scaffold. (**B**) SEM examination of the DBM scaffold. (SEM, scanning electron microscopy).

After approximately 10 days of processing, the decellularized femoral head bones were tailored into cylinders and submitted for scanning electron microscopy (SEM) examination. The SEM images revealed that soft tissue was removed completely, and there was no obvious cellular debris residue on the surface of the DBM scaffold. The cylindrical DBM scaffolds were identified as a porous material (a size range of 200 ~ 400 μm), with a similar microstructure to the native cancellous bone tissue (Fig. 1).

### Animal care and gross observations

**Figure 2:**
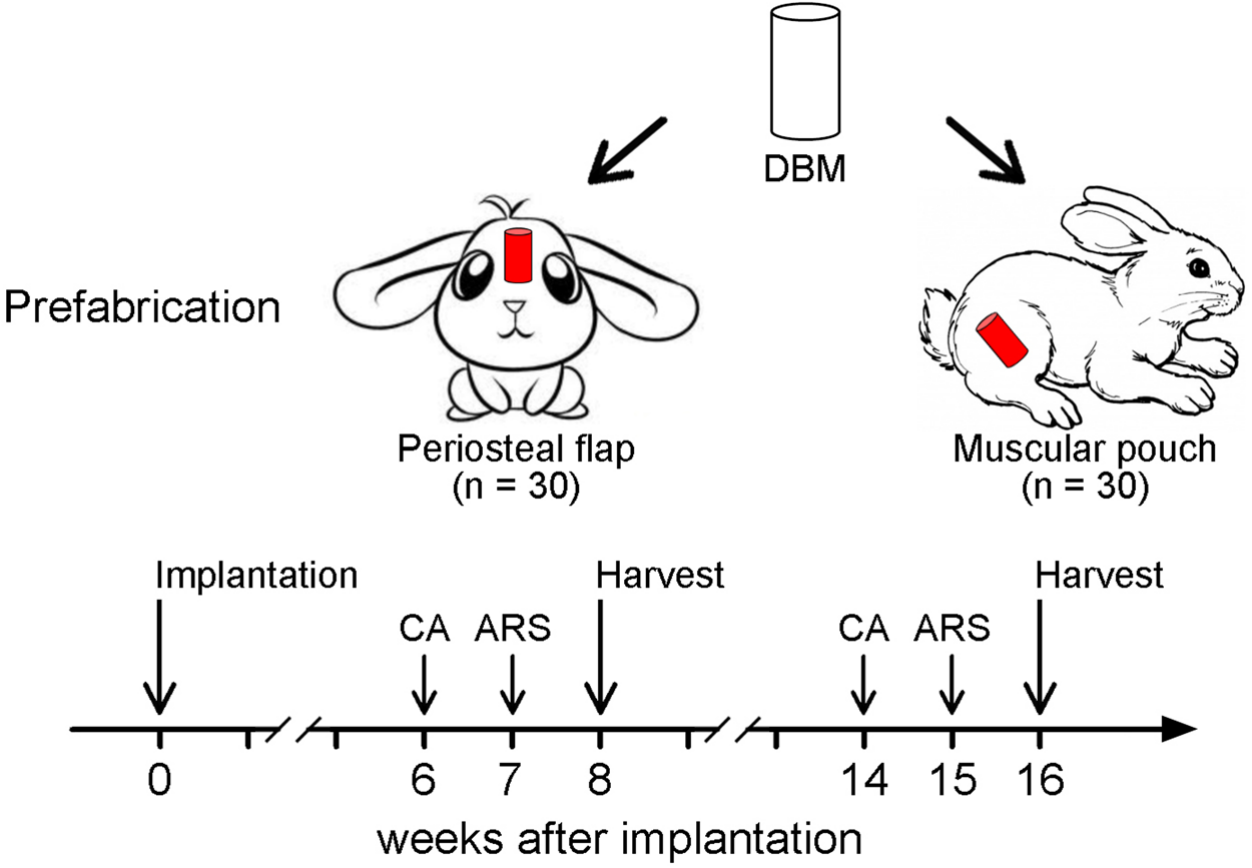
Diagram of the experimental group and time course of the experimental protocol. (**A**) In the experimental group, the DBM was wrapped with pedicled periosteal flaps; in the control group, the DBM was implanted in muscular pouches. (**B**) After 8 and 16 weeks of *in vivo* cultivation, prefabricated bone grafts were harvested. One and 2 weeks before sample harvests, CA and ARS, respectively, were intramuscularly injected into rabbits to label the newly mineralized bone tissue. (CA, Calcein; ARS, Alizarin Red S).

**Figure 3:**
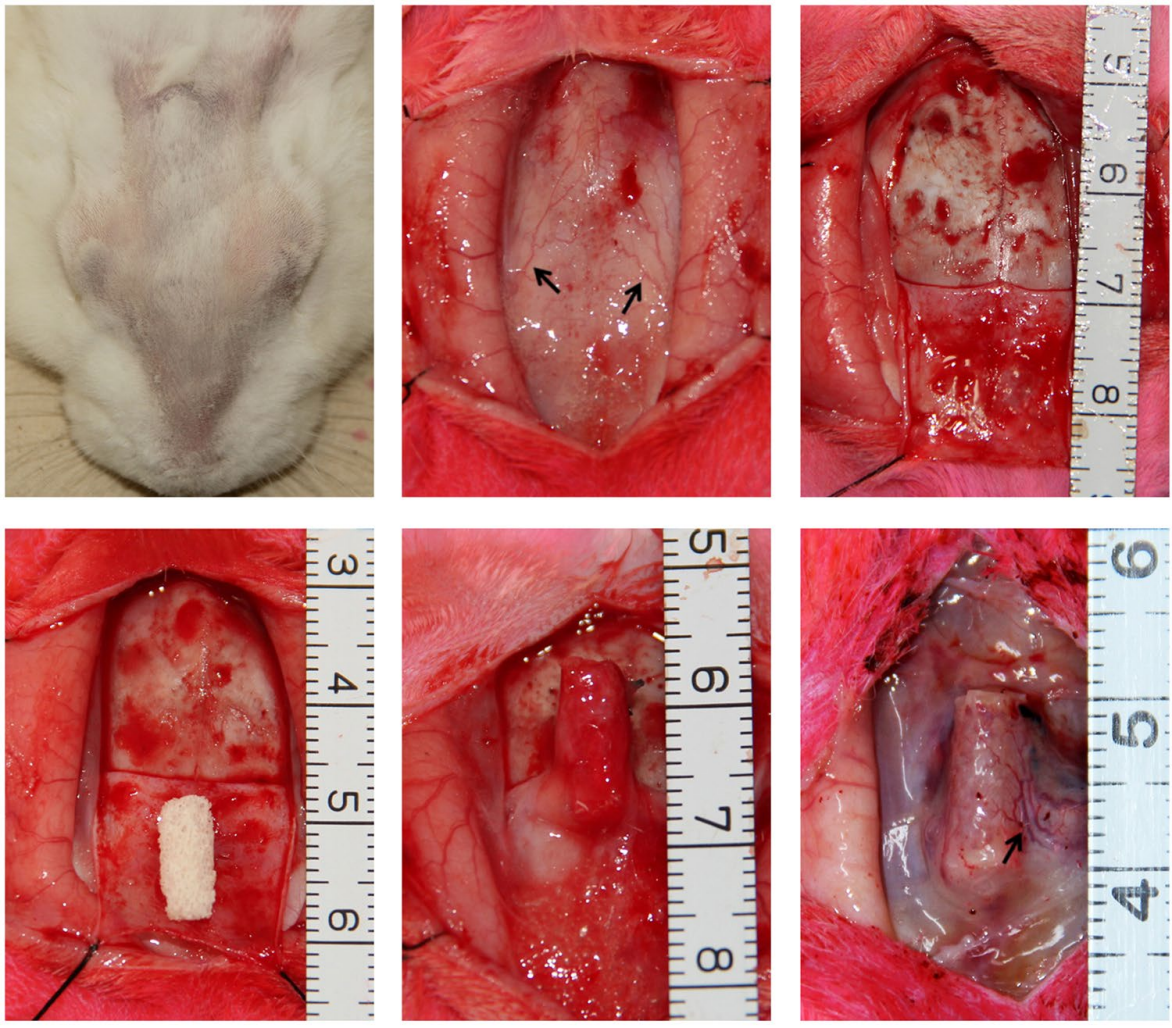
Surgical procedures of bone graft prefabrication using a pedicled periosteal flap and DBM scaffold. (**A**,**B**) Parietal fur was shaved, and a 4-cm vertical incision was made on the top of the skull to expose the skull periosteum. The supraorbital vessels (black arrows) could be identified with the naked eye. (**C**–**E**) A pedicled periosteal flap based on the supraorbital vessels was elevated using a periosteal detacher. A DBM scaffold was wrapped in the pedicled periosteal flap to form a periosteum-DBM construct. (**F**) Implantation of the construct 16 weeks later; the supraorbital vessels were still visible in the periosteal flap (black arrow).

**Figure 4:**
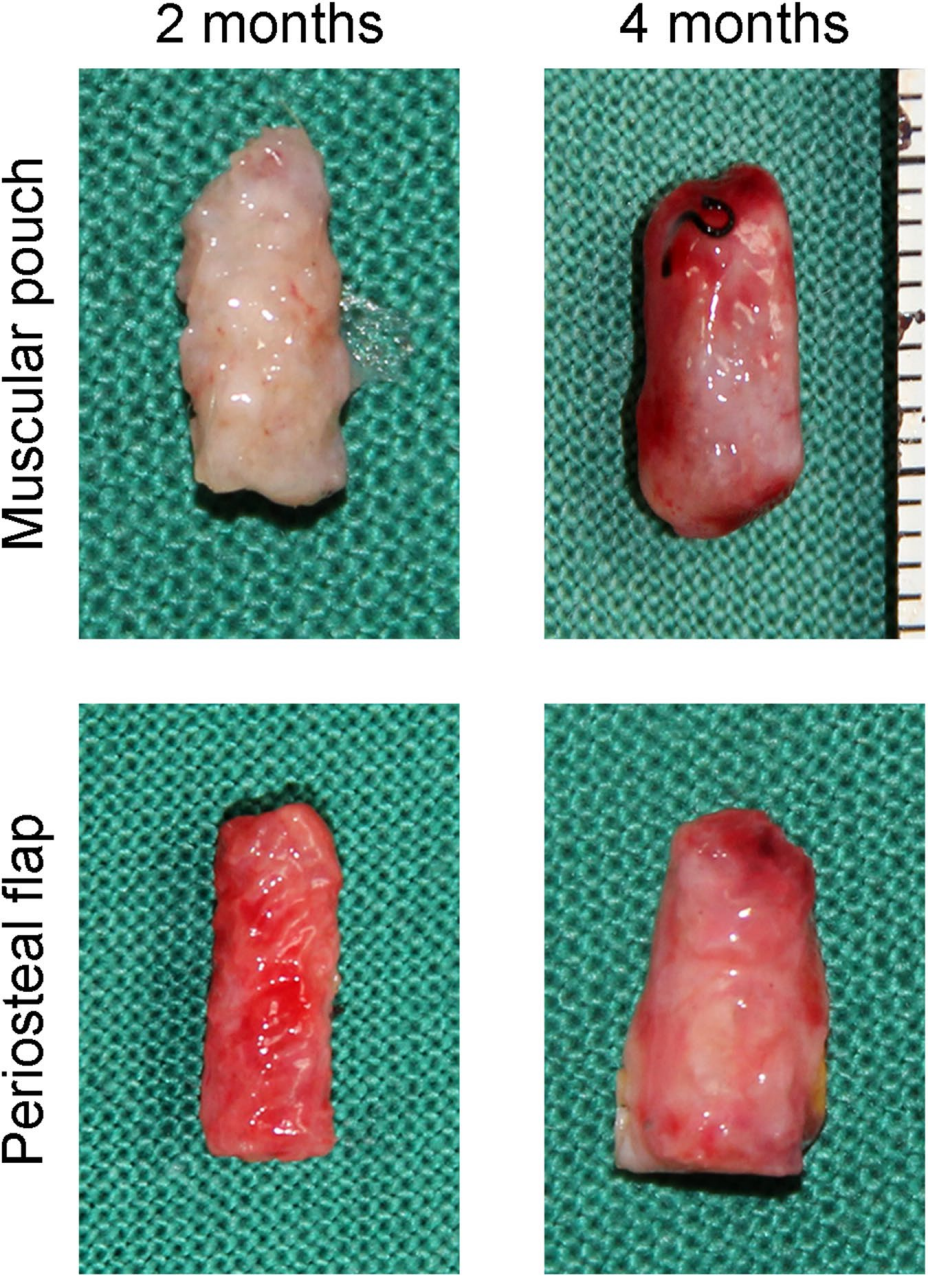
Gross view of the prefabricated bone grafts. (**A**) Bone prefabricated using the muscular pouch strategy. (**B**) Bone grafts prefabricated using the pedicled periosteal flap strategy.

The experimental group and time course of the experimental protocol are shown in Fig. 2. Overall, 60 DBM scaffolds were implanted in the periosteal flap group and the muscular pouch group (n = 30, respectively). Bone graft prefabrication surgeries were successfully performed in all rabbits (Fig. 3). All DBM scaffolds were well tolerated in the implantation sites with no adverse events. Of the 60 implanted DBM scaffolds, 30 were explanted after 8 weeks and 30 were explanted after 16 weeks. Gross examination of the bone grafts showed that all the bone graft samples were wrapped with muscular tissue or periosteum, and no obvious necrosis was observed (Fig. 4). Bone grafts from each group and each time point were divided into 3 groups: 5 were submitted for mechanical analysis, 5 were submitted for decalcified sections, and 5 were submitted for hard tissue sections.

### MicroCT scanning and quantitative evaluation

**Figure 5:**
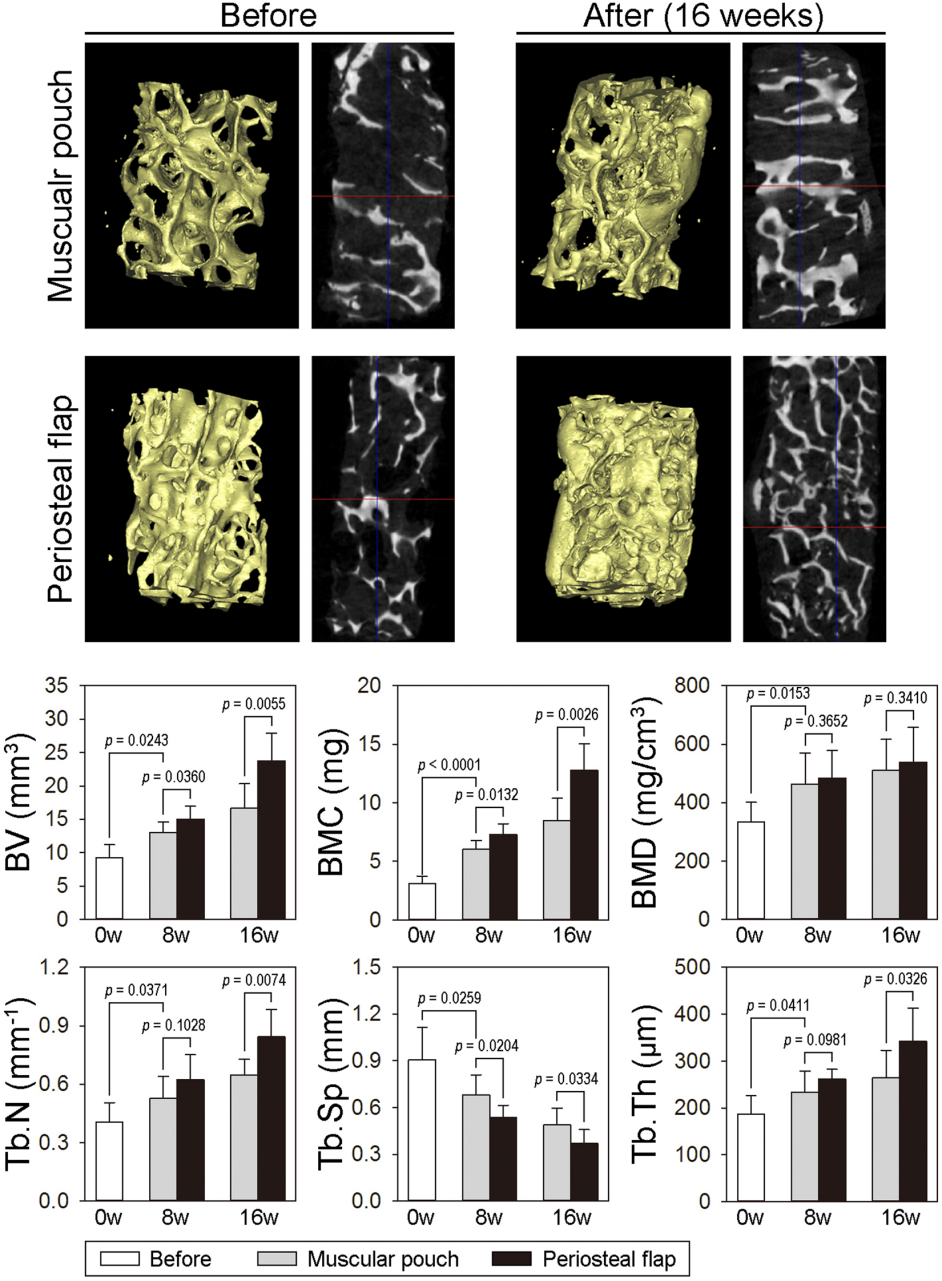
MicroCT scanning of the prefabricated bone grafts. (**A**) Representative 3D reconstruction images and 2D images of the same DBM scaffold before implantation and 16 weeks after implantation. (**B**) Morphometric analysis of BV, BMC, BMD, Tb.N, Tb.Sp, and Tb.Th as determined by microCT for each group and time point compared to the DBM scaffolds or the muscular pouch group (n = 10). (BV, bone volume; BMC, bone mineral content; BMD, bone mineral density; Tb.N, trabecular number; Tb.Sp, trabecular separation; Tb.Th, trabecular thickness).

All bone graft specimens were submitted for microCT analysis before histological analysis. Three dimensional (3D) reconstructive images were harvested from two dimensional (2D) microCT scanning images. Representative preoperative and 16 weeks postoperative images of two DBM scaffolds, which were assigned to the periosteal flap group and the muscular pouch group, respectively, are shown in Fig. 5A. These 3D and 2D microCT scanning images preliminarily demonstrated more trabecular number and increased mineralized volume occurred after 16 weeks of *in vivo* cultivation compared with the preoperative images.

To quantitatively evaluate the increased bone mass, bone morphological parameters, including bone volume (BV), bone mineral content (BMC), bone mineral density (BMD), trabecular number (Tb.N), trabecular thickness (Tb.Th), and trabecular separation (Tb.Sp), were analyzed. As shown in Fig. 5B, *in vivo* cultivation resulted in significantly increased bone mass in a time-dependent course manner. In the muscular pouch group, the BV increased 1.40-fold at 8 weeks and 1.79-fold at 16 weeks, and the BMC increased 1.94-fold at 8 weeks and 2.73-fold at 16 weeks. In the periosteal flap group, the BV increased 1.62 folds at 8 weeks and 2.56 folds at 16 weeks, and the BMC increased 2.34-fold at 8 weeks and 4.11-fold at 16 weeks. More importantly, the increases of BV, BMC, and BMD were statistically significant in both the muscular pouch group and periosteal flap group (*p* < 0.05, compared with the bone morphometry data of the DBM scaffold before implantation), indicating that the *in vivo* bioreactor strategy induced a considerable amount of new bone formation on the DBM scaffolds. To compare the bone regeneration efficiency of the two *in vivo* bioreactor strategies, the bone morphological data were analyzed between the two groups. Compared with the muscular pouch group, the periosteal flap group showed superiority in terms of a larger BV (1.16-fold at 8 weeks, *p* = 0.036; 1.43-fold at 16 weeks, *p* = 0.006) and higher BMC (1.21-fold at 8 weeks, *p* = 0.013; 1.50-fold at 16 weeks, *p* = 0.003). Furthermore, the bone grafts from the periosteal group showed more Tb.N and higher Tb.Th but smaller Th.Sp. than that from the muscular pouch group at 16 weeks. These data suggest that the periosteal flap induced more mineral tissue formation on the surface of the DBM scaffold.

### Histological and histomorphometric examinations

**Figure 6:**
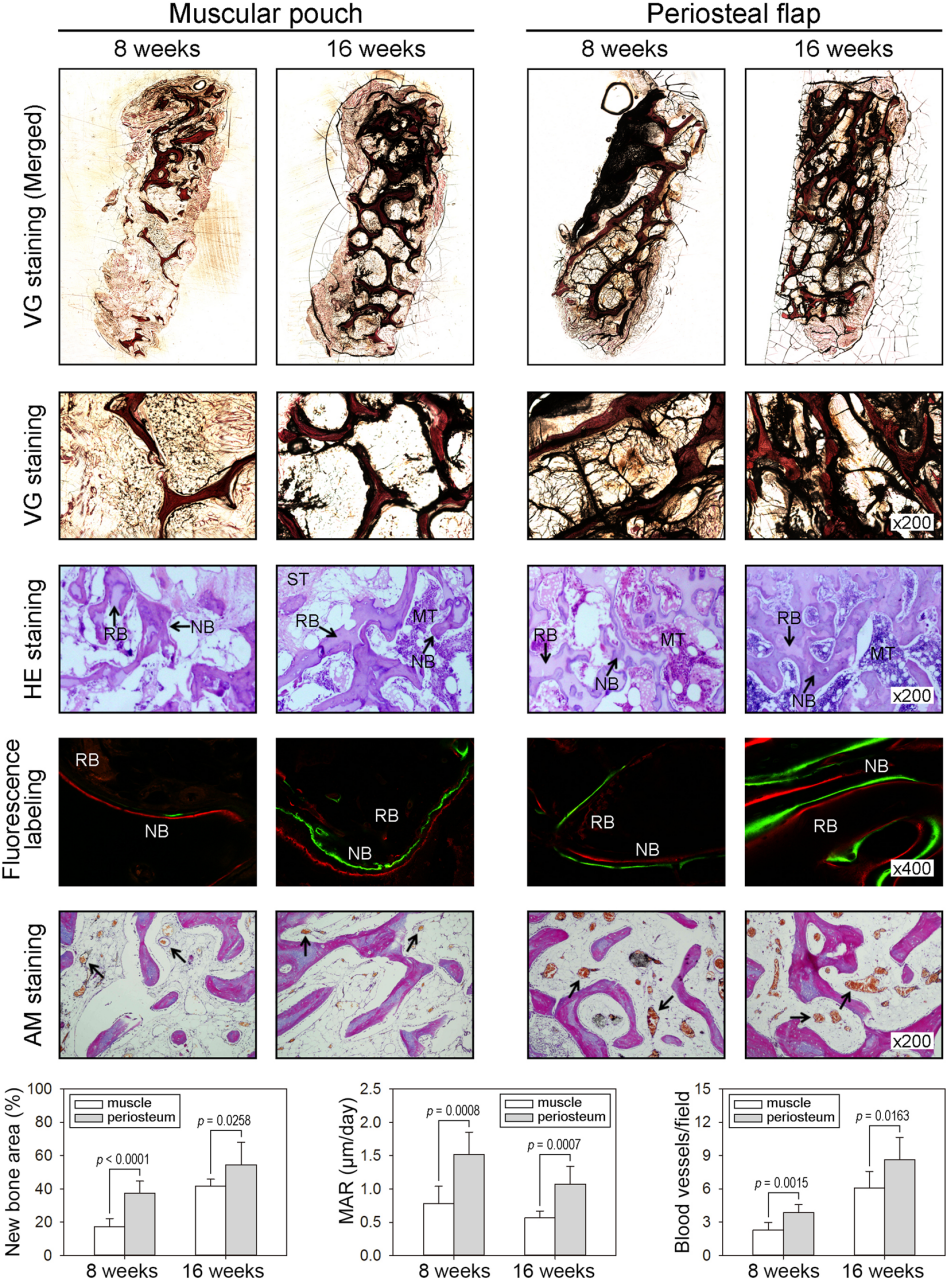
Histological examination of the prefabricated bone grafts. (**A**,**B**) The undecalcified bone grafts from each group and time point were sectioned and stained with VG picrofuchsin. Bone tissue is shown in red. (**C**) The decalcified sections of bone grafts were stained with HE. The residual DBM scaffolds were stained light pink, and the new bone tissue was stained deep pink. (**D**) The decalcified sections of bone grafts from each group and time point were stained with AM. (**E**) The new bone tissue in undecalcified sections was labeled with CA and ARS at 6, 7, 14, and 15 weeks post-implantation. (**F**) Quantitative analysis of the new bone area in HE-stained sections (n = 5). (**G**) Quantitative analysis of blood vessel densities in AM-stained sections (n = 5). (**H**) Quantitative analysis of the mineral apposition rate in undecalcified sections (n = 5). (RB, residual bone tissue; NB, new bone tissue; ST, soft tissue; MT, mesenchymal tissue; compared with the muscular pouch group).

The persistence of residual implanted DBM scaffolds and newly formed bone tissue within the regenerated bone grafts was examined using histological and histomorphometric analysis. All histologic sections were taken from the longitudinal view of generated bone grafts. The merged van Gieson’s (VG) staining images demonstrated the microstructure of the bone trabeculae and the shape of the generated bone grafts (Fig. 6A), and the magnified VG staining images showed newly formed collagen tissue in all specimens (Fig. 6B). The newly formed osteoid matrix was distinguished from the residual implanted DBM scaffolds by the distinct mottled staining pattern as evidenced by hematoxylin and eosin (HE) staining (Fig. 6C). Bony structures appeared as dense pink structures. However, the residual implanted DBM scaffolds were stained light pink, and the newly formed bone tissue, which was located on the surface of the residual DBM scaffolds, appeared as deep pink. More importantly, the areas of newly formed bone tissue in the specimens from each group and time point were significantly different. The area of new bone in the periosteal flap group was 2.40-fold larger at 8 weeks (*p* < 0.001) and 1.45-fold larger at 16 weeks (*p* = 0.026) than that in the muscular pouch (Fig. 6F), indicating that new bone formation was clearly higher in the periosteal flap group than in the muscular pouch group at both 8 and 16 weeks.

### Sequential fluorochrome-labeling analyses

To evaluate the bone regeneration rate of the two strategies, Alizarin Red S (ARS) and Calcein (CA) were intramuscularly injected into rabbits one and two weeks before bone graft explanation, respectively, and then, confocal laser scanning microscopy (CLSM) was applied to observe sequential fluorochrome-labeling (Fig. 2B). As shown in the CLSM images, active new bone formation and mineralization were observed in bone grafts from both the muscular pouch group and the periosteal flap group during the whole period of *in vivo* cultivation (Fig. 6D). However, as shown in the 8-week sections, weak CA and ARS deposition was observed in the bone grafts from the muscular pouch group, as demonstrated by a smaller fluorochrome-labeled area than that from the periosteal flap group. Quantitative analysis of the distance of the two fluorochrome labels showed that the mineral apposition rate (MAR) was 0.78 μm/day in the muscular pouch group and 1.52 μm/day in the periosteal flap group at 6–7 weeks. At weeks 14 and 15, the rabbits received CA and ARS injections, respectively, for the second time, and the labeled area was significantly larger in the periosteal flap group than in the muscular pouch group (*p* < 0.001 at both 8 and 16 weeks). The MAR was 0.57 μm/day in the muscular pouch group and 1.07 μm/day in the muscular pouch group at 14–15 weeks (Fig. 6G). These data revealed that (1) the periosteal flap strategy possesses a faster bone regeneration rate than in the muscular pouch strategy, and (2) the bone regeneration rate becomes slow, which may be due to increased bone remodeling.

### Angiogenesis analyses

Analysis of the vascular components was shown in the Azan-Mallory (AM) staining images, in which the blood vessel walls were identified as blue collagen fibers and red blood cells were stained orange. The bone graft sections from both groups showed a considerable amount of blood vessels (Fig. 6E). However, statistical analysis of the blood vessel densities showed dramatically higher angiogenesis in the periosteal flap group than in the muscular pouch group (*p* = 0.002 at 8 weeks, *p* = 0.016 at 16 weeks). Quantitative analysis of the blood vessels showed that the blood vessel densities in the muscular pouch group were 2.30 per field at 8 weeks and 6.07 per field at 16 weeks, and the blood vessel densities in the periosteal flap group were 3.87 per field at 8 weeks and 8.62 per field at 16 weeks (Fig. 6H). These data concordantly suggest that the periosteal flap strategy possesses a stronger angiogenic capacity than in the muscular pouch strategy.

### Biomechanical analyses

**Figure 7:**
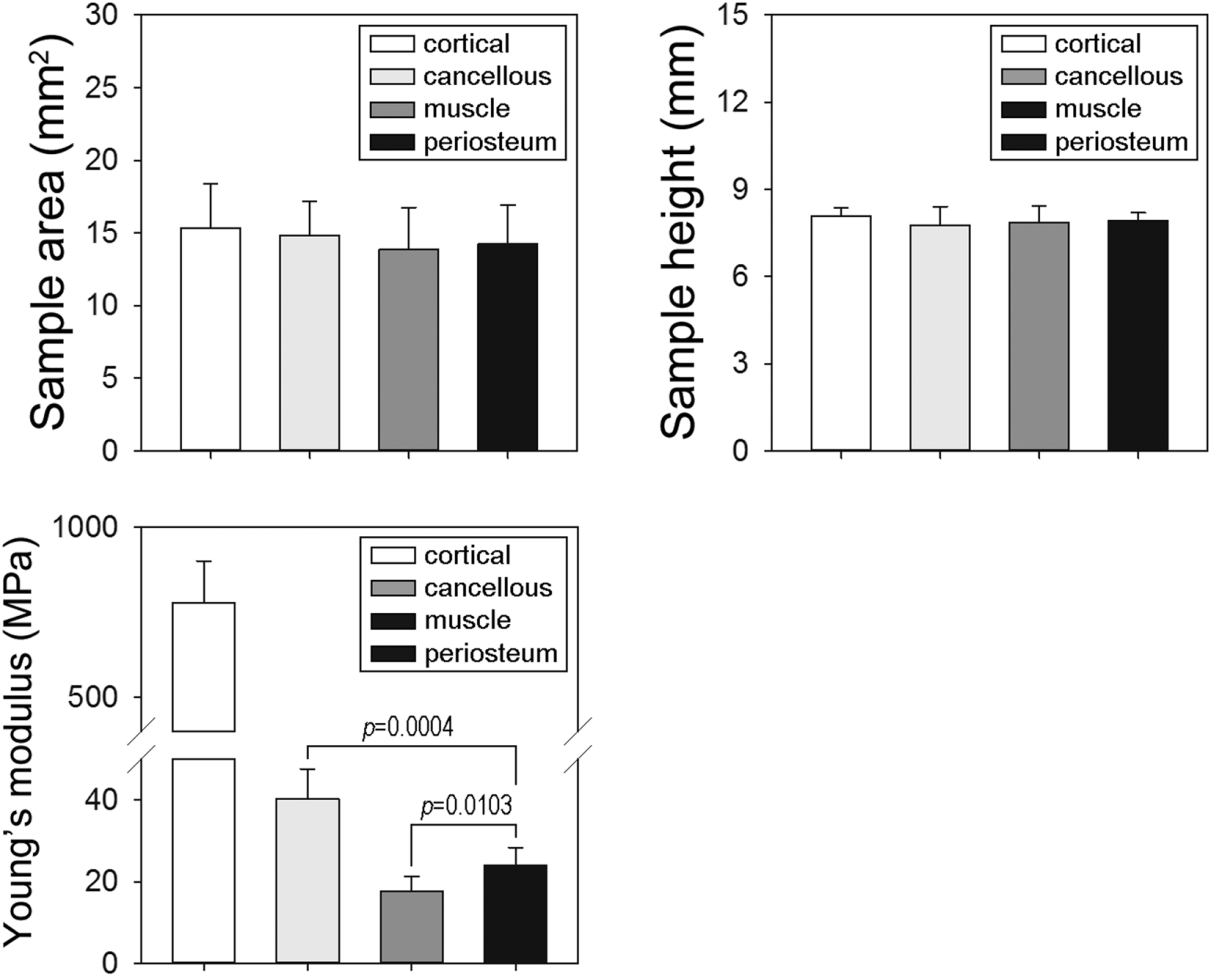
Mechanical properties of the prefabricated bone grafts. (**A**,**B**) The cross-sectional area and height of all samples was measured using Image-Pro 5.0 software (n = 5). (**C**) Young’s modulus of all samples compared with the muscular pouch group or the native cancellous bone samples (n = 5).

To evaluate the biomechanical characteristics of the prefabricated bone grafts, freshly harvested bone grafts were submitted for comparison with autologous native cancellous (n = 5) and cortical bone samples (n = 5) by Young’s modulus (Fig. 7). Before the biomechanical test, the cross-sectional area (*p* = 0.805) and height (*p* = 0.692) of each sample were examined and showed no significant differences (Fig. 7A,B). Young’s modulus of prefabricated bone grafts was significantly lower than the native cancellous bone, especially the cortical bone samples (Fig. 7C). More precisely, the bone grafts from the periosteal flap group possessed approximately 60% of Young’s modulus of cancellous bone samples but only 3% of cortical bone samples. Most importantly, bone grafts from the periosteal flap group showed a significantly higher Young’s modulus than in the muscular pouch group (up to 1.36-fold, *p* = 0.01). The periosteal flap strategy considerably enhanced the biomechanical properties of the prefabricated bone grafts over in the muscular pouch strategy.

## Discussion

The regeneration of vascularized functional bone grafts with minimum donor site morbidity and maximum size for large bone defect reconstruction would be a tremendous advancement for reconstructive surgery. This study offers a proof of concept that a vascularized functional bone graft can be efficiently prefabricated with a pedicled periosteal flap and a DBM scaffold. The pedicled periosteal flap strategy yielded higher osteoinductive and angioinductive properties and considerable enhancement of biomechanical properties of the prefabricated bone grafts compared with the muscular pouch strategy. Thus, the technique using a pedicled periosteal flap as an *in vivo* bioreactor to cultivate a functional bone graft is a feasible and promising approach for bone graft prefabrication.

Generation of vascularized bone tissue following the *in vivo* bioreactor principle was already proposed since 2005^21,22^. Following the *in vivo* bioreactor principle, the *in vivo* bioreactor is a vascular territory as well as a regenerative niche for vascularization, regeneration, and remodeling of prefabricated bone grafts^23^. Here, we describe the use of a pedicled periosteal flap as an *in vivo* bioreactor to regenerate functional bone grafts, in contrast to the use of a muscular pouch or flap, which is one of the most commonly used strategies due to its rich capillary and nerve network^24,25^. Theoretically, the most important consideration for an *in vivo* bioreactor strategy is the tissue type surrounding the bioreactor, which may directly affect the interaction with the implanted construct, recruitment of autologous cells, reestablishment of a functional neurovascular network, and the results of bone graft prefabrication. Compared with the muscle tissue, several significant features of the periosteum support this as an excellent strategy for bone graft prefabrication: a niche for skeletal progenitor/stem cells^26^; an abundance of molecular signals^27^; increased nutrient-rich blood supply and acting as a natural barrier membrane to prevent soft-tissue invasion^28^. Therefore, the rabbit skull periosteal flap pedicled on the supraorbital vessel was chosen as an *in vivo* bioreactor, and the femoral muscle was chosen as a control strategy. To quantificationally compare osteoinductive capacity of the pedicled periosteal flap strategy and the muscular pouch strategy, bone mass of the same decellularized bone was evaluated by microCT scanning at reoperation and postoperation (Fig. 5). After a period of *in vivo* cultivation, significant increases of BV and BMC were observed in a time-dependent manner in the both groups. However, the increase bone mass in the bone grafts from the periosteal flap group was obviously larger than that from the muscular pouch group. Comprehensive with the results from fluorescence labeling (Fig. 6D and G) and HE staining (Fig. 6C and F), the bone grafts prefabricated from the pedicled periosteal flaps showed larger bone mass and a faster bone regeneration rate.

Normally, periosteum was used as a free periosteum graft^29^ or a random periosteal flap^18^ to wrap or cover a bioscaffold and support bone regeneration. However, these strategies produced limited clinical success; only one clinical case regarding mandibular defect reconstruction was reported^30^, indicating that the current periosteum-based bone graft prefabrication strategies need improvement. To access the capacity of revitalization and revascularization, an AVB or AVL was inserted into a bioscaffold or composite construct in some reports^31,32^. We previously used a tibial periosteum in combination with a separated saphenous AVB to serve as an *in vivo* bioreactor for bone graft prefabrication^33^. In this study, we further improved this strategy by creating a pedicled periosteal flap based on its own supraorbital vessels to serve as an *in vivo* bioreactor. Theoretically, this pedicled periosteal flap can be considered as a random periosteal flap in combination with an AVB or an AVL, both of which are well-established bone graft prefabrication strategies^23^. The AVB or AVL can transport progenitor/stem cells, cytokines, oxygen, and nutrients from the whole body; remove waste products from the cells; and result in excellent vascularization and osteogenesis of the scaffold^34,35^. In this study, the bone grafts from the pedicled flaps showed accelerated vascularization, especially at the early stage of bone graft prefabrication (8 weeks postoperation, Fig. 6H) than that from the muscular pouches, indicating a stronger angiogenic capacity of the pedicled flaps. Furthermore, the current proposed strategy process promising clinical translational prospect. A pedicled periosteal flap can be transferred as a pedicled composite tissue flap or a free flap for optical reconstruction, which may greatly expand the armamentarium of reconstructive options.

The current reported periosteum-based strategies for bone graft prefabrication have several limitations. One of the most important considerations is the lack of an adequate donor site to offer a large periosteal flap for large or geometry-customized bone graft prefabrication. In this study, skull periosteum and its accompanying supraorbital vessels were chosen as the *in vivo* bioreactor due to several surgical advantages, such as large size for donation, thickness for elevation, and its ease of exposure. However, the rib periosteum is the most commonly used periosteum donor site in large animal models and human cases. Inspirited by promising results in a sheep model^36,37^, Cheng *et al*. used the rib periosteum as an *in vivo* bioreactor to support the regeneration of bone tissue in a chamber filled with morcellized autografts^30^. Although a solid bone graft with dimensions of 1 × 1 × 5 cm was observed extending from the periosteal flap after 8 weeks of implantation, the size and stability of the prefabricated bone grafts were still limited; only a height of 5 mm was augmented on a native mandibular defect after transplantation. The limited mandibular augmentation indicated an obvious bone absorption in the regenerated bone graft at the receipt site. By piecing two or more pieces of the adjacent rib periosteal flap together, a customized *in vivo* bioreactor could be constructed with different shapes and sizes for clinical bone graft prefabrication under various bone defect situations.

The increased biomechanical properties of bone grafts from the periosteal flap group demonstrated that the pedicled periosteal flap possesses excellent osteoinductive properties and is also superior to a muscular pouch for bone modeling and remodeling. Although there were lower biomechanical characteristics than the native cortical and cancellous bone samples, a longer time for *in vivo* prefabrication, such as six months, may allow the development of more mature biomechanical characteristics. However, functional bone defect reconstruction requires that the bone grafts possess similar biomechanical properties as much as possible. In a recent study, the thoracodorsal trunk and latissimus dorsi muscle were used as *in vivo* bioreactors and β-TCP as a bioscaffold to prefabricate bone grafts in a sheep model^15^. After six months of *in vivo* cultivation, the bioartificial vascularized bone grafts revealed approximately 20% of intrinsic stiffness to autologous native cancellous bone samples^35^, and this strategy was successfully applied in a human case for a large-size mandibular defect reconstruction^38^. In this study, with the help of the pedicled periosteal flap strategy, the bone grafts revealed approximately 60% of intrinsic stiffness to native cancellous bone samples, suggesting an analog application for bone defect reconstruction. However, further studies need to be performed on a critical-size bone defect reconstruction model, such as a radial bone defect model or a mandibular defect model, to evaluate the mechanical function and osteointegrative capacity of prefabricated bone grafts under different bone graft requirement conditions.

To the best of our knowledge, this is the first comparative study to evaluate the osteoinductive and angioinductive capacity of a pedicled periosteal flap strategy and muscular pouch strategy for bone graft prefabrication. Furthermore, this study is also the first to use a pedicled periosteal flap as an *in vivo* bioreactor to prefabricate bone grafts. Our findings support the superiority of the pedicled periosteal flap over the muscular pouch and reveal a promising strategy for regenerating functional bone grafts of clinically applicable shapes and sizes. However, limited by animal size and vascular caliber, two issues were not well-addressed in this study. First, a control group using a random periosteal flap as an *in vivo* bioreactor is needed to highlight the advantages of the pedicled periosteal flap. Second, additional studies are needed on a large animal model to further evaluate the feasibility of transferring the prefabricated bone graft as a pedicled flap or a free flap and the functional characteristics of the prefabricated bone grafts.

## Materials and Methods

### Preparation and characterization of the DBM scaffolds

The DBM scaffolds were derived from eleven donated, human femoral head bones (n = 11 donors) during artificial femoral head replacement, and informed consent was obtained in accordance with the Declaration of Helsinki. DBM scaffolds were prepared as previously reported^39^. All methods regarding human tissue samples in this study were approved and conducted in accordance with the Laboratory Animal Research Committee of Shanghai Jiao Tong University School of Medicine. Briefly, after removal of residual soft tissues, the cancellous bones of the femoral heads were trimmed to cylinders with a diameter of 4 mm and length of 10 mm using a trephine. After repeated washing and processes were performed, including degreasing, decalcification, and removal of non-collagen protein were performed, SEM (PhilipsXL-30, Amsterdam) scanning was performed to determine DBM microstructure and porosity as previously described^39^. Finally, the prepared scaffolds were sterilized by gamma-radiation (Zhejiang YinDu Ltd, China) before use.

### Surgical procedure of bone graft prefabrication

All animal procedures in this study were approved and conducted in accordance with the Laboratory Animal Research Committee of Shanghai Jiao Tong University School of Medicine. In total, 60 New Zealand white rabbits with an average body weight of 2.5 ± 0.4 kg (Shanghai Experimental Animal Center, China) were randomly allocated to two groups: the muscular pouch group (*n* = 30) and the periosteal flap group (*n* = 30). The rabbits were anesthetized by face mask induction with 3.5% halothane followed by intubation with 3.5% halothane in oxygen. The parietal and femoral fur was shaved, the operative area was sterilized with 10% povidone-iodine, and the animal was placed in a supine position. For the periosteal flap group, a 4-cm vertical incision was made on the top of the skull to expose the skull periosteum. By careful dissection and hemostasis, a pedicled periosteal flap (1.5 × 1.5 cm) based on the supraorbital vessels was elevated using a periosteal detacher. Then, a DBM scaffold was wrapped in this periosteal flap using 5–0 nylon to form a periosteum-DBM construct. The construct was sutured to adjunctive tissue to minimize movement. The incision was closed with 4–0 nylon. For the muscular pouch group, a 3-cm vertical incision was made on the right femur to expose the femoral muscle. By careful dissection and hemostasis, a muscular pouch was made in the femoral muscle, and a DBM scaffold was inserted into the muscular pouch. The muscular pouch was closed with 5–0 nylon, and the incision was closed using 4–0 nylon. Overall, 60 DBM scaffolds were used in 60 rabbits. Each rabbit was administered 20 mg of enrofloxacin intraperitoneally. The animals were monitored daily for postoperative complications.

### Sequential fluorescent labeling

One and 2 weeks before the harvest of bone graft specimens, the rabbits were intramuscularly injected with CA (Sigma, USA, 20 mg/kg body weight) and ARS S (Sigma, 30 mg/kg body weight), respectively. Mineralized tissues were observed by means of sequential fluorescent labeling using a CLSM (Leica Microsystems Ltd, Germany). Excitation/emission wavelengths were as follows: 488/517 nm (CA, green) and 543/617 nm (ARS, red). The bone formation indices were evaluated using Image-Pro 5.0 software (Media Cybernetics, Rockville, MD, USA), and the MAR (μm/day) was measured.

### Histologic and histomorphometric analyses

The bone specimens harvested at 8 and 16 weeks postoperatively were used for histological and histomorphometric analyses. Half of the specimens were fixed in 4% paraformaldehyde for 2 days. Then, the specimens were decalcified in 20% ethylenediaminetetraacetic acid for 30 days and dehydrated, embedded in paraffin, and sectioned. Sections were stained with HE and AM for the evaluation of bone structures and blood vessels as previously described^39^. Meanwhile, the other half of the specimens was dehydrated in a graded alcohol series and then embedded in polymethylmethacrylate. After hardening, longitudinal sections were cut into 150–200 μm slices using a microtome (Leica), glued onto a plastic support, and then polished to a final thickness of approximately 50 μm. First, the sections were examined for fluorescent labeling. Then, the sections were stained with VG picrofuchsin to evaluate new bone formation. The area of new bone formation was quantitatively evaluated in four random sections using Image-Pro 5.0 software (Media Cybernetics).

### MicroCT scanning

All bone graft specimens were imaged and analyzed with a high-resolution microCT (SkyScan1176, Skyscan, Belgium) with 80 kV source voltage, 100 mA source current, 35 μm pixel size, 180° rotation, 0.2 seconds exposition time, frame averaging 20, and aluminum-copper filters. On average, 1260 slides per sample were reconstructed using NRecon software (CTAn1.8, Skyscan, Belgium). To compare the changes of morphological parameter data, the DBM scaffolds were scanned preoperatively and postoperatively. To measure newly formed bone, a circular area of pre-defined size was selected as the region of interest (ROI) in the two-dimensional images. The pixel zone representing ossification in the defined ROI was then reconstructed in 3D by creating a volume of interest in the lower and upper ranges of the threshold using grayscale units. After using CTAn 1.8 on each reconstructed BMP file, BV, BMD, Tb.Th, Tb.N, and Tb.Sp were obtained using a CT-analyzer in direct 3D based on a surface-rendered volume model. In addition, total BMC was calculated by multiplying BV and BMD.

### Biomechanical analyses

Four months after bone graft prefabrication, compression tests were performed on six prefabricated bone grafts from the periosteal flap group, six prefabricated bone grafts from the muscular pouch group, eight native cancellous bone samples from the iliac crest, and eight native cortical bone samples from the radius to evaluate the biomechanical properties. These samples were chosen as controls because of their similar cross-sectional area and the fact that both bone qualities (cancellous and cortical bone) were used for bone defect reconstruction. Prior to mechanical testing, the test samples were cut into cylinders with the same diameter as the prefabricated bone grafts (4 mm) and a height of 8 mm. Then, the cross-sectional area and height of the test samples were measured using Image-Pro 5.0 software (Media Cybernetics). The samples were tested under uniaxial compression using a biomechanical analyzer (Instron-8874, Canton, MA) according to a previously described protocol^39^.

### Statistical analyses

All the above data were analyzed as the means ± standard deviations. One-way analysis of variance (ANOVA) and Tukey’s post-test multiple comparison tests were used to determine significance between experimental groups. Student’s t test was used to determine significance between two groups. *P* values < 0.05 were considered statistically significant. IBM SPSS Statistics V20 was used for statistical analyses.
